# 1263. The Experience of an Antimicrobial Stewardship Program at a Tertiary Referral Hospital in a Low/Middle Income Country

**DOI:** 10.1093/ofid/ofad500.1103

**Published:** 2023-11-27

**Authors:** Tamara Abdallah, Nisrine Haddad, Nesrine Rizk, Rony Zeenny, Souha S Kanj

**Affiliations:** American University of Beirut Medical Center, Beirut, Beyrouth, Lebanon; American University of Beirut Medical Center, Beirut, Beyrouth, Lebanon; American University of Beirut, Beirut, Beyrouth, Lebanon; American University of Beirut medical Center, Beirut, Beyrouth, Lebanon; American University of Beirut Medical Center, Beirut, Beyrouth, Lebanon

## Abstract

**Background:**

Antimicrobial Stewardship Programs (ASP) play a crucial role in optimizing antimicrobial utilization and limiting the use of broad-spectrum antibiotics, to reduce antimicrobial resistance. During the COVID-19 pandemic, there was a noticeable increase in antimicrobial consumption globally. The ASP at the American University of Beirut Medical Center (AUBMC) began formal operations in January 2019. In this study, our aim is to evaluate the activities of the ASP -since implementation and during the pandemic- at our institution, a tertiary referral hospital in Beirut.

**Methods:**

The ASP team performed handshake stewardship rounds and daily reviews of antimicrobial use. It provided educational activities in a multi-disciplinary approach in addition to implementing a carbapenem sparing strategy and ongoing monitoring of antibiotic consumption. This study is a retrospective review of those activities. We compiled the ASP recommendations from January 2019 until December 2021. Data collected included the number of ASP recommendations and their sub-categories in addition to acceptance rates.

**Results:**

We recorded a total of 9922 recommendations during this three-year period. Recommendations aiming at reducing antimicrobial consumption were the most common (42%), and included: duration of therapy, de-escalation, and duplicate coverage (Figure 1). Rates of acceptance of these recommendations were 88%, 92% and 95% in 2019, 2020 and 2021 respectively. The number of recommendations increased during the pandemic and coincided with COVID-19 surges in Lebanon (figure 2). There was also an increase in the acceptance rate of recommendations to reduce antibiotic consumption (Figure 3) over this period.

**Figure 1.**
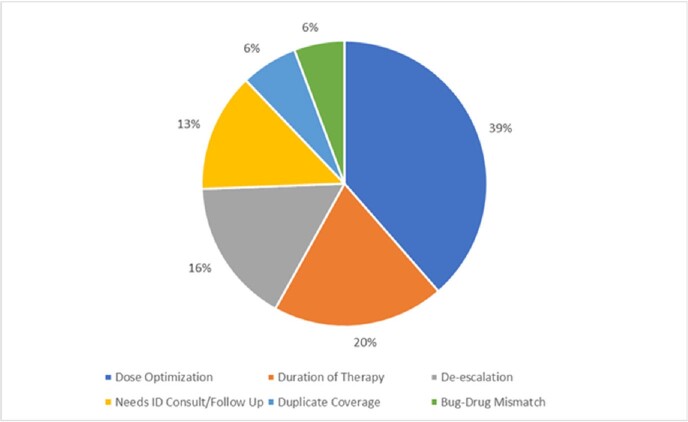
Distribution of ASP Interventions per sub-Category

**Figure 2.**
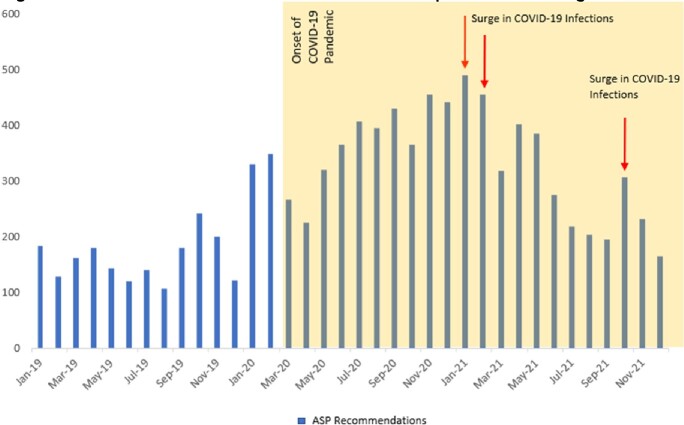
Number of ASP Recommendations pre- and During COVID-19

**Figure 3.**
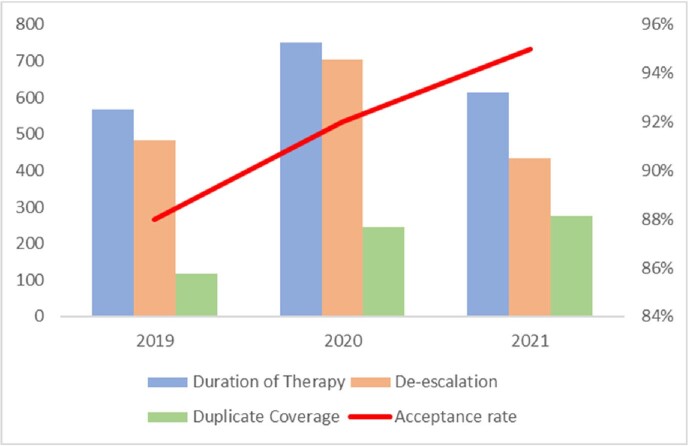
ASP recommendations to Reduce Antibiotic Consumption and Acceptance Rate

**Conclusion:**

Despite the challenges imposed by the pandemic and the involvement of the ASP in the COVID-19 response, the team maintained its efforts and involvement to mitigate antibiotic misuse. The results are encouraging and attest to the important role of ASP in driving culture change regarding the judicious use of antimicrobials even during a pandemic.

**Disclosures:**

**Souha S Kanj, MD**, Gilead: Advisor/Consultant|Menarini: Advisor/Consultant|MSD: Advisor/Consultant|Pfizer: Advisor/Consultant

